# Dropped head syndrome due to myogenic atrophy --- a case report of surgical treatment

**DOI:** 10.1186/1746-1596-6-9

**Published:** 2011-01-19

**Authors:** Michihisa Zenmyo, Masahiko Abematsu, Takuya Yamamoto, Yasuhiro Ishidou, Setsuro Komiya, Kosei Ijiri

**Affiliations:** 1 Orthopaedic Surgery, Graduate School of Medical and Dental Sciences, Kagoshima University, Kagoshima, Japan

## Abstract

We report a case of a 69-year-old man with dropped head syndrome associated with isolated neck extensor myopathy (INEM). Over a period of 2 years, he exhibited progressive inability to lift his chin off his chest, resulting in the dropped head position that impaired his activities of daily living. He had a disturbed gait with severe imbalance of spinal alignment. Computed tomography revealed osseous contracture of cervical vertebral bodies in flexed position. Anterior combined posterior reconstruction surgery yielded a successful outcome in his activities of daily living, including his walking balance of spinal alignment. Pathologic study confirmed myogenic atrophy in the cervical extensor muscles. We suggest that consideration for surgical management should be given to dropped head syndrome especially due to INEM.

## Background

Dropped head syndrome (DHS) is characterized by severe paravertebral extensor muscle weakness, resulting in chin-on-chest deformity [1]. Various conditions associated with DHS have been reported, including neuromuscular diseases, e.g. myasthenia gravis, motor neuron disease, congenital myopathy, chronic inflammatory demyelinating polyneuropathy, and mitochondriopathy [2]. Some of these conditions that present with DHS are progressive and systemic, and have a grave prognosis, such as amyotrophic lateral sclerosis [3]. Others have a benign clinical course without generalized neuromuscular disorder, and are diagnosed as isolated neck extensor myopathy (INEM).

The indications for surgical treatment of INEM are controversial, since these conditions have only rarely been described [4]. We report here a case of DHS due to INEM that was successfully treated surgically, with a review of the literature.

### Case Presentation

**Figure 1:**
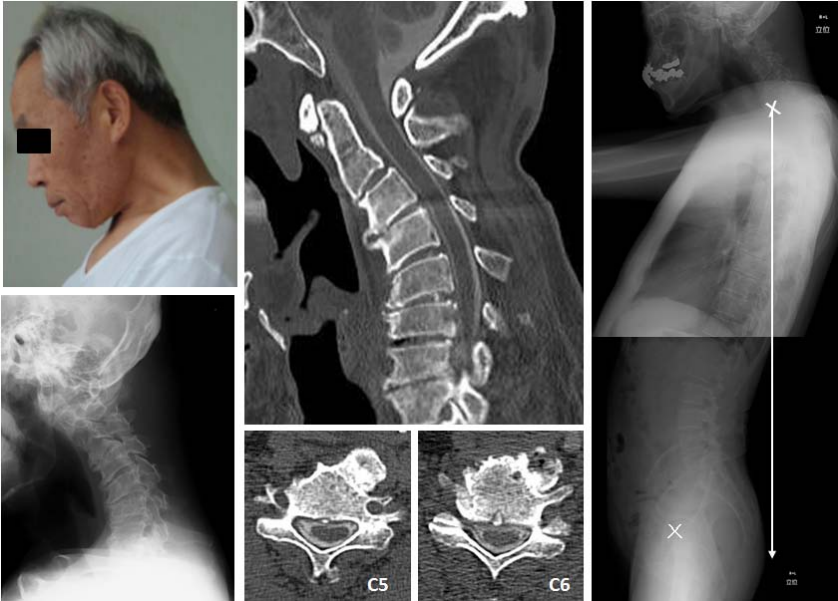
**Dropped head deformity before surgery**. Radiographs showing degenerative kyphoscoliosis with ossification of the anterior longitudinal ligament. Impairment in total spinal alignment due to cervical kyphosis.

Over a period of 2 years, a previously healthy 69-year-old man exhibited progressive inability to lift his chin off his chest. The dropped head position severely impaired his activities of daily living. When he visited our institution, he complained of severe neck pain without neurological abnormalities such as motor weakness or sensory disturbance. He had no family history of DHS. No systemic risk factors, such as diabetes, vascular disorders, history of smoking, were reported. He was working as an office worker for 25 years, without potential professional hazard for DHS. A plain radiograph revealed cervical degenerative kyphoscoliosis with ossification of the anterior longitudinal ligament (Figure 1). Electrodiagnostic study demonstrated polyphasic motor unit potentials (MUP) but no fibrillation potentials in the trapezius muscle. Both the amplitude and duration of voluntary MUP of the trapezius muscle was normal. Resting MUP was not detected due to continuous fibrillation in the splenius capitus muscle. All laboratory findings including creatine kinase level were normal.

Anterior release of vertebral column through C3-6 was performed as the first stage of surgery. Correction of kyphotic deformity was partially achieved using a Halo vest fixator. No neurological deterioration was observed during this procedure. Two weeks after the first stage of surgery, posterior correction and fusion with spinal instrumentation (Mountainer: Depuy Spine Co. Ltd., USA) and anterior bone grafting from C3 to C6, using a free fibula but without the usage of plating, were performed. Finally, cervical kyphotic deformity was corrected from 56° to 14°. After fixation using cervical brace for 3 months, solid bone fusion was confirmed using CT scan.

**Figure 2:**
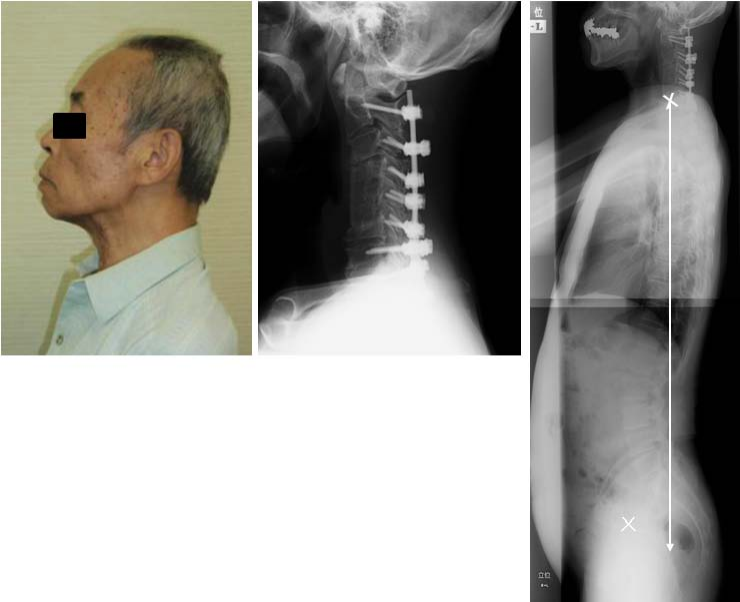
**Improvement of dropped head deformity and total spinal alignment**.

Postoperatively, the patient exhibited improvement of head positioning while standing and walking, without neck pain. Plain lateral radiography in standing position 4 months after surgery revealed improvement of sagittal balance and pelvic tilt compared to preoperative results (preoperative C7 plumb line -85.1 mm → postoperative C7 plumbline -11.7 mm, preoperative pelvic tilt 22° → postoperative pelvic tilt 14°), with significant improvement in overall spinal alignment (Figures 1, 2). The patient reported a dramatic improvement in his quality of life, enabling him to perform the daily activities of eating, reading, standing and walking without difficulty.

**Figure 3:**
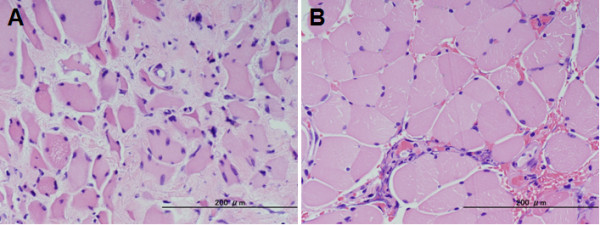
**Histological examination. **(A) H.E. staining of trapezius muscle, showing myogenic atrophy. (B) H.E. staining of sternohyoid muscle, showing normal pattern.

On microscopic examination of the trapezius muscle, variability in fiber size of striated muscle was observed, with scattered intrafibrous nuclei. No group atrophy or ragged-red fibers were found. Fibrous degeneration was pronounced among these striated fibers. Neural fibers were observed around the striated muscle fibers without degeneration (Figure 3A). On the other hand, no significant pathological findings were observed in the sternohyoid muscle samples (Figure 3B). These findings indicated that myogenic atrophy of the cervical extensor muscles was the primary etiology, and not secondary muscle atrophy.

## Conclusions

While some cases of DHS are progressive and systemic, with a grave prognosis, others present with INEM in the absence of generalized neuromuscular disorder and have a benign clinical course [5].

One possible cause of INEM in the present case is mitochondrial disease [6]. However, it has been reported that the paraspinal cervical muscles develop pathological abnormalities with increasing age and that both ragged-red fibers and accumulation of mitochondria are frequent findings in aging cervical muscles [7]. Myasthenia gravis (MG) must be considered as another possible cause of INEM. The clinical history of patients affected by MG is usually characterized by weakness and fatigability of muscles on repeated activity that improve after rest. They usually present not only difficulties of holding their heads upright, but also weakness of legs and ptosis, diplopia and slurring of speech. However, D'Amelio et al. recently described an unusual case of MG, presenting the isolated weakness of neck extensor muscles [8]. A Tensilon test or edrophonium test is essential for the diagnosis of MG, however, the present case was not examined with these tests since the clinical feature was not typical for MG.

Since the number of reported cases is small, it is still controversial whether surgical treatment is indicated for drooped head syndrome. One case of failure after surgical treatment with posterior correction and fusion without anterior reconstruction has been reported [9]. The authors mentioned the risk of implant pullout and failure to achieve adequate fixation of instrumentation in elderly patients due to osteoporosis. As reported here, we recommend anterior bone grafting combined with fusion with posterior instrumentation for DHS. For patients with anterior and posterior fibrous contractures, anterior release combined with posterior release is necessary. Two staged surgery was safe to observe not only neurological deterioration after correction but also activities of daily life in new position. Posterior instrumentation of pedicle screws and lateral mass screws was necessary to get final correction after posterior release of contracted facet joints.

We have reported here a rare case of DHS due to INEM. Anterior combined posterior reconstruction surgery yielded a successful outcome. Pathologic study revealed myogenic atrophy in the cervical extensor muscles, though the pathogenesis of this case remains unclear.

## Consent

Written informed consent was obtained from the patient for publication of this case report and any accompanying images. A copy of the written consent is available for review by the Editor-in-Chief of this journal.

## Competing interests

The authors declare that they have no competing interests.

## Authors' contributions

ZM, MA, YI carried out the immunohistochemical studies. TY, KI participated in the surgery and the design of the study, and KI contributed in the statistical analysis. SK and KI conceived the study and drafted the manuscript. All authors read and approved the final manuscript.
